# Forecasting tumor and vasculature response dynamics to radiation therapy via image based mathematical modeling

**DOI:** 10.1186/s13014-019-1446-2

**Published:** 2020-01-02

**Authors:** David A. Hormuth, Angela M. Jarrett, Thomas E. Yankeelov

**Affiliations:** 1 Oden Institute for Computational Engineering and Sciences, The University of Texas at Austin, 201 E. 24th Street, POB 4.102, 1 University Station (C0200), Austin, TX USA; 2 Livestrong Cancer Institutes, The University of Texas at Austin, Austin, TX USA; 3 Departments of Biomedical Engineering, The University of Texas at Austin, Austin, TX USA; 4 Departments of Diagnostic Medicine, The University of Texas at Austin, Austin, TX USA; 5 Departments of Oncology, The University of Texas at Austin, Austin, TX USA

**Keywords:** DW-MRI, DCE-MRI, Glioma, Diffusion, Mathematical modeling

## Abstract

**Background:**

Intra-and inter-tumoral heterogeneity in growth dynamics and vascularity influence tumor response to radiation therapy. Quantitative imaging techniques capture these dynamics non-invasively, and these data can initialize and constrain predictive models of response on an individual basis.

**Methods:**

We have developed a family of 10 biologically-based mathematical models describing the spatiotemporal dynamics of tumor volume fraction, blood volume fraction, and response to radiation therapy. To evaluate this family of models, rats (*n* = 13) with C6 gliomas were imaged with magnetic resonance imaging (MRI) three times before, and four times following a single fraction of 20 Gy or 40 Gy whole brain irradiation. The first five 3D time series data of tumor volume fraction, estimated from diffusion-weighted (DW-) MRI, and blood volume fraction, estimated from dynamic contrast-enhanced (DCE-) MRI, were used to calibrate tumor-specific model parameters. The most parsimonious and well calibrated of the 10 models, selected using the Akaike information criterion, was then utilized to predict future growth and response at the final two imaging time points. Model predictions were compared at the global level (percent error in tumor volume, and Dice coefficient) as well as at the local or voxel level (concordance correlation coefficient).

**Result:**

The selected model resulted in < 12% error in tumor volume predictions, strong spatial agreement between predicted and observed tumor volumes (Dice coefficient > 0.74), and high level of agreement at the voxel level between the predicted and observed tumor volume fraction and blood volume fraction (concordance correlation coefficient > 0.77 and > 0.65, respectively).

**Conclusions:**

This study demonstrates that serial quantitative MRI data collected before and following radiation therapy can be used to accurately predict tumor and vasculature response with a biologically-based mathematical model that is calibrated on an individual basis. To the best of our knowledge, this is the first effort to characterize the tumor and vasculature response to radiation therapy temporally and spatially using imaging-driven mathematical models.

## Background

Radiation therapy has been a nearly ubiquitous treatment option for cancers for over a century [1]. During that time, there has been steady improvements in treatment delivery driven by increased understanding of radiation biology and engineering advancements in delivery technology. A prime example is for the treatment of gliomas, where complete resection is often practically impossible [2, 3]—necessitating adjuvant radiation (and/or chemo-) therapy to treat residual disease. In this setting, developments in radiation therapy technology has facilitated the delivery of more precise irradiation techniques to optimize the dose delivered to the tumor while simultaneously minimizing exposure to healthy tissue. Even with these advances, prognosis for malignant gliomas is overwhelming poor with response to standard therapies typically resulting in disease progression in 7 to 10 months [2]. Emerging imaging [4] and modeling approaches [5–8], however, may facilitate the improvement of radiation therapy through the assessment of intratumoral heterogeneity, the identification of tumor radiosensitivity, and through in silico trials to optimize therapeutic regimens (e.g., dosing and scheduling) for an individual subject.

Anatomical imaging approaches such as contrast-enhanced magnetic resonance imaging (MRI), play a crucial role in identification of treatment volumes and the assessment of response [9]. However, anatomical imaging does not assess physiological or functional properties of the tissue that might be relevant to tumor and radiation biology. Quantitative imaging techniques such as diffusion weighted (DW-) MRI and dynamic contrast enhanced (DCE-) MRI can provide functional information characterizing changes in tumor cellularity and tissue perfusion, respectively [10]. Both DW-MRI and DCE-MRI have shown promising potential by providing early imaging biomarkers of response in glioblastoma [11, 12] and have been widely used in other cancers [13–15]. The quantitative and non-invasive nature of both DW-MRI and DCE-MRI makes these techniques well-suited for mathematical modeling of tumor growth as they can provide the 3D distribution of the tumor cells and vasculature before, during, and after treatment. These temporally defined data sets allow for model initialization and calibration, both of which are required for making tumor specific predictions.

We (and others [6, 16–22];) have developed a series of increasingly comprehensive biophysical mathematical models of tumor and vasculature growth [5, 23–25] to provide individualized forecasts of response to radiation therapy using non-invasive quantitative imaging data. Our overall hypothesis, is that biologically-relevant models, informed and calibrated from tumor-specific imaging data, may lead to the early prediction of response, significant improvements in tumor control through the pre-treatment optimization of therapy regimens, and the adaptation of treatment regimens by accounting for the spatiotemporal variations in tumor response [26]. Thus, we seek to incorporate the effects of radiation therapy spatially on cellularity and vascularity.

Historically, the linear quadratic [27] (LQ) model has played a fundamental role in the design and delivery of radiation therapy plans. More recently, there has been an increased interest in the development of tumor (or patient) specific approaches to characterize and/or predict response to radiation therapy [8, 28–31]. In the pre-clinical setting, we previously developed [5] a model of response to radiation therapy consisting of immediate (i.e., instantaneous reduction in tumor cells) and long-term (reduced proliferation) effects of radiation therapy. In that study, we demonstrated that combining both immediate and long-term effects outperformed models consisting of single effects. Additionally, we have previously demonstrated that both DW-MRI and DCE-MRI data could be used to initialize and calibrate a biophysical model of tumor growth and angiogenesis in the absence of treatment that resulted in accurate volume and voxel-wise predictions of tumor and blood volume fractions [25]. At the clinical level, one approach by Rockne et al. [6] leveraged anatomical MRI and hypoxia-sensitive positron emission tomography images collected in glioblastoma multiforme patients to determine patient-specific radiosensitivity parameters. Rockne et al. demonstrated that incorporation of hypoxia-mediated resistance resulted in more accurate predictions of the bulk tumor volume. Prokopiou et al. [8] proposed the use of the proliferation saturation index or simply the ratio of the pre-treatment tumor volume to a patient-specific carrying capacity to predict and personalize radiotherapy fractionation. In an in silico clinical trial, Prokopiou et al. demonstrated that patients with proliferation saturation indices ranging from 0.45 to 0.9 were more likely to benefit from the proposed hyperfractionation protocol as opposed to the standard fractionation protocol. These promising approaches represent real progress towards the development of model-based adaptive radiation therapy. However, we posit that tumor cellularity and volumetric measures alone may be insufficient to capture the spatially and biologically heterogenous response to radiation therapy.

In this contribution, we extend our image driven tumor induced angiogenesis model to incorporate the effects of radiation therapy on cell death and the net proliferation rate. We first develop a family of 10 biologically-relevant models to investigate different approaches to characterize the response of the tumor and vasculature to radiation therapy. We then calibrate all 10 models using quantitative DW-MRI and DCE-MRI data collected in a murine model of glioma growth on an animal-specific basis. The most parsimonious and well calibrated model is then chosen by a statistical model selection scheme. Finally, we assess the model’s ability to accurately predict response in volume and voxel-wise measures of tumor and blood volume fraction as observed in post-treatment MRI measures.

## Methods

### Biophysical model of tumor and vasculature growth

Our mathematical model is built upon the well-studied reaction-diffusion model framework that has been extensively applied to pre-clinical [23, 32, 33] and clinical models [29] of glioma growth. In a single species model (i.e., considering only tumor cells of one type) the reaction diffusion model describes the spatial and temporal change in tumor cell number due to proliferation (i.e., the reaction term) and due to the outward movement (i.e., the diffusion term) of tumor cells. In our previous efforts, we extended the standard reaction-diffusion model to incorporate the effect of local tissue stress on tumor cell diffusion [24] as well as characterize the spatial-temporal evolution of both tumor cells and vasculature [25]. We present the salient features of this model system here, while Table 1 summarizes the model parameters and variables and their sources. (A more detailed description can be found elsewhere [24, 25, 34].) The spatial and temporal evolution of the tumor cell fraction is given by Eq. (1):
1

**Table 1:** Model parameters and variables

Parameter or variable	Interpretation	Source
${\phi}_T\left(\overline{x},t\right)$	Tumor cell volume fraction	Measured from DW-MRI
${\phi}_V\left(\overline{x},t\right)$	Blood volume fraction	Measured from DCE-MRI
*k*_*p*,*T*_	Tumor cell proliferation rate	Calibrated
${\theta}_{T,V}\left(\overline{x},t\right)$	Combined ${\phi}_T$ and ${\phi}_V$ carrying capacity	Calculated
${\theta}_T\left(\overline{x},t\right)$	Tumor cell carrying capacity	Calculated Eq. [4]
*D*_*T*,*0*_,*D*_*V*,*0*_	${\phi}_T$ and ${\phi}_V$ diffusion coefficients in absence of mechanically coupling	Calibrated
*G*	Shear modulus	Literature [34]
*v*	Poisson’s Ratio	Literature [34]
*λ*_1_	Coupling Constants	Calibrated
*λ*_2_	Coupling Constants	Set to 1
${\phi}_{V,\mathrm{thresh}}$	Threshold on ${\phi}_V$ for carrying capacity to decrease	Calibrated
*θ*_max_	Max carrying capacity	Calibrated
*θ*_min_	Minimum value for carrying capacity	Assigned from DW-MRI
*k*_*p*,*V*_	Vasculature proliferation rate	Calibrated
*k*_*d*,*V*_	Vasculature death rate	Calibrated
*d*	Distance top the periphery of the tumor	Calculated
*θ*_*V*_	Maximum blood volume	Assigned from DCE-MRI
*α*_*I*_	Treatment efficacy (immediate effect)	Calibrated
*α*_*LT*_	Treatment efficacy (long term effect)	Calibrated
*Dose*	Treatment dose (in Gy)	Assigned
*α*	Radiosensitivity Parameters	Calibrated
*β*	Radiosensitivity Parameter	Assigned
$\overline{\phi_{V, pre- treatment}}$	Average pre-treatment${\phi}_V\left(\overline{x},t\right)$	Calculated

where ${\phi}_T\left(\overline{x},t\right)$ is the tumor cell fraction at three-dimensional position $\overline{x}$ and time *t*, ${D}_T\left(\overline{x},t\right)$ is the tumor cell diffusion coefficient, ${\theta}_{T,V}\left(\overline{x},t\right)$ is the summation of tumor and the blood volume fraction carrying capacities, ${\phi}_V\left(\overline{x},t\right)$ is the blood volume fraction, *k*_*p,T*_ is the tumor cell proliferation rate, and ${\theta}_T\left(\overline{x},t\right)$ is the tumor cell carrying capacity (i.e., the maximum packing fraction that a voxel can functionally support). Tumor cell diffusion is affected by both local tissue mechanical properties, via ${D}_T\left(\overline{x},t\right)$, and by the space occupied by tumor associated vasculature. The effect of local tissue properties is incorporated by exponentially dampening ${D}_T\left(\overline{x},t\right)$ as the local mechanical stress increases according to:

2 $$ {D}_T\left(\overline{x},t\right)={D}_{T,0}\exp \left(-{\lambda}_1\cdot {\sigma}_{vm}\left(\overline{x},t\right)\right), $$

where *D*_*T,0*_ represents the uninhibited tumor cell diffusion coefficient, *λ*_1_ is the stress-tumor cell diffusion coupling constant, and ${\sigma}_{vm}\left(\overline{x},t\right)$ is the von Mises stress which reflects the total stress experienced for a given section of tissue. The ${\sigma}_{vm}\left(\overline{x},t\right)$ is determined by solving for tissue displacement, $\overrightarrow{u}$, using the linear elastic, isotropic equilibrium equation (Eq. (3)):

3 $$ \nabla \cdot G\nabla \overrightarrow{u}+\nabla \frac{G}{1-2v}\left(\nabla \cdot \overrightarrow{u}\right)-{\lambda}_2\nabla {\phi}_T\left(\overline{x},t\right)=0, $$

where *G* is the shear modulus,*ν* is the Poisson’s Ratio, and *λ*_2_ is the second coupling constant. Literature values are used to assign tissue specific [34] *G* and *v*. ${\theta}_T\left(\overline{x},t\right)$, and ${\theta}_{T,V}\left(\overline{x},t\right)$, are temporally and spatially varied in response to changes in ${\phi}_V\left(\overline{x},t\right)$ using the following linear relationship:

4 $$ {\theta}_T\left(\overline{x},t\right)=\Big\{{\displaystyle \begin{array}{cc}{\theta}_{\mathrm{max}}& {\phi}_V\left(\overline{x},t\right)\ge {\phi}_{V, thresh}\\ {}{\theta}_{\mathrm{min}}+{\phi}_V\left(\overline{x},t\right)\left(\frac{\theta_{\mathrm{max}}-{\theta}_{\mathrm{min}}}{\phi_{V, thresh}}\right)& {\phi}_V\left(\overline{x},t\right)<{\phi}_{V, thresh},\end{array}} $$

where *θ*_min_ to *θ*_max_ represents the range of expected carrying capacity values, and *ϕ*_*V*, *thresh*_ represents a critical value for ${\phi}_V\left(\overline{x},t\right)$ that would begin to change the number of cells a voxel can support. *θ*_min_ is assigned as the lowest volume fraction within the tumor.

The spatial-temporal evolution of ${\phi}_V\left(\overline{x},t\right)$, is described with a similar reaction-diffusion model consisting of a diffusion, angiogenesis, and death terms as shown in Eq. (5):
5

where ${D}_V\left(\overline{x},t\right)$ is the vascular diffusion coefficient, *k*_*p,V*_ is the vascular growth rate, *θ*_*V*_ is the blood volume fraction carrying capacity, *d* is a normalized parameter describing the distance to the periphery of the tumor, and *k*_*d,V*_ is the vascular death rate. ${D}_V\left(\overline{x},t\right)$ is also coupled to local tissue stress as in a similar fashion as ${D}_T\left(\overline{x},t\right)$. *d* ranges from 1 for voxels at the periphery of the tumor to 0 for voxels furthest from the periphery.

### Modeling response to radiation therapy

We assume the response to radiation therapy is observed over two-time frames: immediate and long term. First, for the immediate response following radiation therapy, some cells die instantaneously due to irreparable damage or early mitotic catastrophe [35, 36]. Second, for long term response following radiation therapy, some cells will have a reduced net proliferation rate due to DNA repair processes or delayed mitotic catastrophe [35, 36]. The immediate effects of radiation therapy are modeled as a direct reduction of ${\phi}_T\left(\overline{x},t\right)$ at the time of radiation therapy:

6 $$ {\phi}_{T, postRT}\left(\overline{x},t\right)={\phi}_{T, preRT}\left(\overline{x},t\right)-{\alpha}_I\cdot {C}_I\cdot {\phi}_{T, preRT}\left(\overline{x},t\right) $$

where ${\phi}_{T, postRT}\left(\overline{x},t\right)$ is the post-radiation therapy value of the tumor cell fraction, ${\phi}_{T, preRT}\left(\overline{x},t\right)$ is the pre-radiation therapy value of the tumor cell fraction, *α*_*I*_ is an treatment efficacy parameter for the immediate effects, and *C*_*I*_ is one of five coupling approaches (described below). The post-radiation effects of ${\phi}_V\left(\overline{x},t\right)$ are handeled in an identical fashion as Eq. (6). The long term effects *R*_*LT*_(*t*) of radiation therapy are incorporated in an amended Eq. (1):
7

Eq. (5) is amended in a similar fashion as Eq. (7). The term *R*_*LT*_(*t*) is a piecewise function represented by Eq. (8):

8 $$ {R}_{LT}(t)=\Big\{{\displaystyle \begin{array}{cc}{\alpha}_{LT}\cdot {C}_{LT}& t\ge {t}_{rt}\\ {}1& t<{t}_{rt}\end{array}} $$

where *α*_*LT*_ is a treatment efficacy parameter, *C*_*LT*_ is one of five coupling approaches (described below), and *t*_*rt*_ is the time that radiation therapy is administered.

To systematically investigate different approaches for characterizing the efficacy of radiation therapy spatially or as a function of treatment dose, we implemented five different coupling approaches as shown in Table 2. The logistic growth approach, *C*_*1*_, assumes the efficacy of radiation therapy decreases as ${\phi}_T\left(\overline{x},t\right)$ approaches ${\theta}_T\left(\overline{x},t\right)$ due to a slower proliferation (and thus less susceptible) cells. The LQ approach, *C*_*2*_, assumes no spatial variance in treatment efficacy, but relates efficacy to the radiosensitivity parameters (*α* and *β*) and the treatment dose (*Dose*). The vascular coupled approach, *C*_*3*_, relates the treatment efficacy spatially to ${\phi}_V\left(\overline{x},t\right)$. Here, we assume areas that are well-vascularized (potentially normoxic) and have increased radiosensivity relative to poorly-vascularized (potentially hypoxic) regions. The oxygen enhanced approach, *C*_*4*_, combines elements from *C*_*2*_ and *C*_*3*_ to spatially vary treatmenty efficacy as a function of ${\phi}_V\left(\overline{x},t\right)$ while also utilizing a common radiobiology adaptation of the LQ model. While ${\phi}_V\left(\overline{x},t\right)$ is not a direct measure of tissue oxygenation, we assume that tissue oxygenation is proportional to the blood volume fraction; thus, when ${\phi}_V\left(\overline{x},t\right)$ is greater than the average pre-treatment ${\phi}_V\left(\overline{x},t\right)$, $\overline{\phi_{V, pre- treatment}}$, the oxygen enhancement ratio (OER) will be greater than 1. Finally, the fifth coupling approach, *C*_*5*_, assumes no spatial variability, and the effect of radiation therapy is evenly applied.

**Table 2:** Radiation therapy efficacy coupling approaches

	Coupling approach	Formula
*C*_*1*_	Logistic	${C}_I={C}_{LT}=1-{\phi}_T\left(\overline{x},t\right)/{\theta}_T\left(\overline{x},t\right)$
*C*_*2*_	Linear quadratic (LQ)	*C*_*I*_ = 1 − exp(−*α* ⋅ *Dose* − *β* ⋅ *Dose*^2^), *C*_*LT*_ = 1 − *C*_*I*_
*C*_*3*_	Vascular	${C}_I=\exp \left(-\left({\theta}_V-{\phi}_V\left(\overline{x},t\right)\right).{\alpha}_3\right),{C}_{LT}=1-{C}_I$
*C*_*4*_	Oxygen enhanced	$$ {\displaystyle \begin{array}{l} OER={\phi}_V\left(\overline{x},t\right)/\overline{\phi_{V, pre- treatment}}\\ {}{C}_I=1-\exp \left( OER\cdot \left(-\alpha \cdot Dos e-\beta \cdot Dos{e}^2\right)\right),{C}_{LT}=1-{C}_I\end{array}} $$
*C*_*5*_	None	*C*_*I*_ = 1, *C*_*LT*_ = 1

The spatial-temporal evolution of ${\phi}_T\left(\overline{x},t\right)$ and ${\phi}_V\left(\overline{x},t\right)$ was deterimined using a finite difference approximation implemented in MATLAB R2018a (Mathworks, Natick, MA) using a fully explicit in time differentiation (time step = 0.01 days) and three dimension in space (Δ*x* = 250 μm, Δ*y* = 250 μm, Δ*z* = 1000 μm) central difference spatial differentiation. No flux (Neumann) boundary conditions were used for ${\phi}_T\left(\overline{x},t\right)$ and ${\phi}_V\left(\overline{x},t\right)$ at the skull boundary. The boundary condition for $\overrightarrow{u}$ was assumed to be zero displacement in the normal direction, while it was assumed that the tissue in the tangential directions was free to move (i.e., slip condition). Complete numerical details on the mechanically-coupled model, Eq. (3), can be found elsewhere [34].

### In vivo experimental methods

All experimental procedures were approved by our Institutional Animal Care and Use Committee. Thirteen female Wistar rats (weights from 240 to 286 g) were anesthetized and inoculated intracranially with 10^5^ C6 glioma cells (obtained from Sigma-Aldrich, St. Louis, MO, USA). Rats were imaged with MRI on days 10, 12, 14, 16.5, 18.5, 20.5, and 22.5. A permanent jugular catheter was placed in each rat up to 48 h prior to their first MRI study. Rats received a single fraction 20 Gy (*n* = 5) or 40 Gy (*n* = 8) of whole brain radiation therapy on day 14.5 using a Therapax DXT 300 x-ray machine (300 kVp/10 mA, Pantak Inc., East Haven, CT, USA) at a dose rate of 2.3 Gy/min. During the irradiation procedure, the animals were anesthetized with a mixture of 2% isoflurane in 98% oxygen, and lead shielding was used to minimize radiation exposure outside of the brain.

**Fig. 1:**
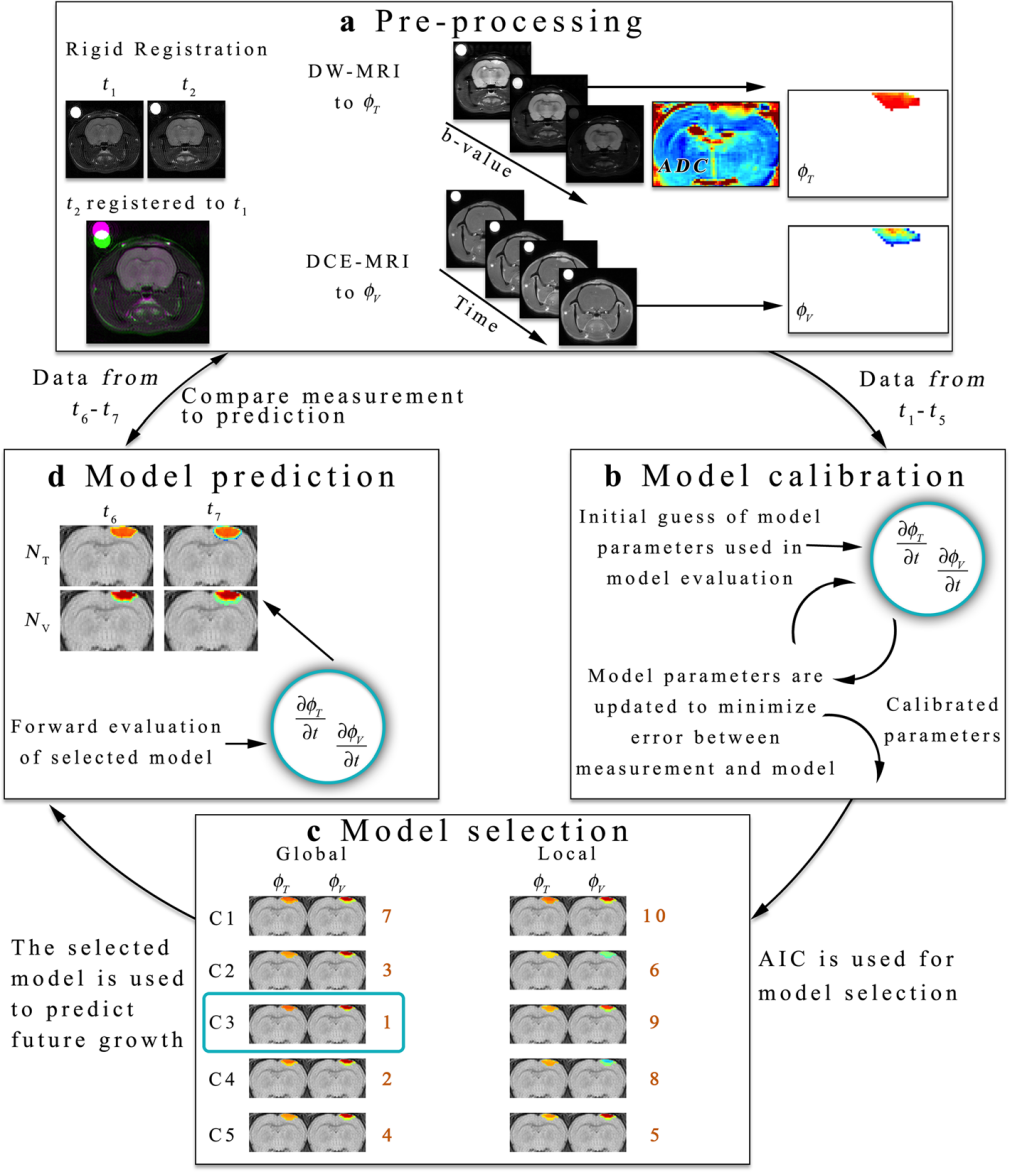
Schematic of experimental and computational methods. Panel **a** shows our model pre-processing work flow. Images are first registered using a rigid registration algorithm. DW- and DCE-MRI are then used to estimate ${\phi}_T\left(\overline{x},t\right)$ and ${\phi}_V\left(\overline{x},t\right)$ at each time point. Using data from *t*_*1*_ through *t*_*5*_, all 10 models are calibrated (panel **b**) to return estimates of the model parameters that minimize the error between the measurement and model. The AIC is then calculated and used to select the most appropriate model (panel **c**) that balances model complexity and model fit. The selected model is then used in a forward evaluation to provide a “forecast” (panel **d**) of future ${\phi}_T\left(\overline{x},t\right)$ and ${\phi}_V\left(\overline{x},t\right)$ at *t*_*6*_ and *t*_*7*_. The predicted ${\phi}_T\left(\overline{x},t\right)$ and ${\phi}_V\left(\overline{x},t\right)$ are then compared directly back to the measurement obtained from DW- and DCE-MRI

MRI data was acquired using a 9.4 T horizontal-bore magnet (Agilent, Santa Clara, CA) with a 38 mm diameter Litz quadrature coil (Doty Scientific, Columbia, SC, USA) over a 32 × 32 × 16 mm^3^ field of view sampled with a 128 × 128 × 16 matrix. Figure 1 summarizes the experimental and computational framework used in this study. Following the initial imaging visit, all subsequent imaging visits were registered using a mutual information based rigid registration algorithm to provide imaging spatial offsets and rotations to minimize retrospective registration [23] (Fig. 1a). The same registration algorithm was used to perform retrospective registration as needed to maximally align the imaging data across time. DW-MRI and DCE-MRI experiments were performed at each visit. For the DW-MRI experiment, a pulsed gradient, fast spin echo sequence with three orthogonal diffusion encoding directions with *b*-values of 150, 500, 1100 s/mm^2^, *TR* = 2000 ms, *TE* = 30 ms, number of excitations = 10, *Δ =* 25 ms, and *δ* = 2 ms. A standard mono-exponential model was fit to the DW-MRI data to estimate the apparent diffusion coefficient (*ADC*) voxel-wise within the tumor. *ADC* measures (Fig. 1a) at each voxel were then used to estimate ${\phi}_T\left(\overline{x},t\right)$ assuming the linear relationship shown in Eq. (9):

9 $$ {\phi}_T\left(\overline{x},t\right)=\left(\frac{AD{C}_w- AD C\left(\overline{x},t\right)}{AD{C}_w- AD{C}_{\mathrm{min}}}\right) $$

where *ADC*_*w*_ is the *ADC* of free water at 37 °C [37], $ADC\left(\overline{x},t\right)$ is the *ADC* value at position $\overline{x}$ and time *t*, and *ADC*_min_ is the minimum *ADC* observed within the tumor regions-of-interest (ROIs) across all animals. We have previously used Eq. (9) to provide non-invasive estimates of tumor cellularity [5, 24, 38–40]; however, we acknowledge that the assumption that all changes in *ADC* are related to changes in cellularity is a simplification of the complexity of the cellular (e.g., cell size and permeability) and tissue properties (e.g., tortuosity) in the presence and absence of treatment that also contributes to changes in *ADC* (see discussion in [5, 25]). For the DCE-MRI experiment, we first collected a *T*_*1*_ map using an inversion-recovery snapshot with *TR* = 5000 ms, *TE* = 3 ms, 8 inversion times logarithmically spaced between 200 and 4000 ms, and two averaged excitations. DCE-MRI data was then acquired using a spoiled gradient echo sequence with *TR* = 45 ms, *TE* = 1.4 ms, two averaged excitations, and a flip angle of 20°. A 200 μL bolus (0.05 mmol kg^− 1^) of Gado-DTPA™ (BioPhysics Assay Lab, Worcester, MA) was injected after 25 image sets were acquired. Relative blood volume fraction, ${\phi}_V\left(\overline{x},t\right)$, was calculated by computing the ratio of the area under the curve for the concentration of the contrast agent in tissue time course to the arterial input function [41] over the first 60 s (Fig. 1a). Tumor ROIs were identified using a relative enhancement map (post-contrast image divided by pre-contrast image) derived from DCE-MRI data.

### Model parameter calibration

In addition to the five coupling scenarios described above (i.e., *C*_*1*_ to *C*_*5*_), we considered two additional parameterization approaches. First, we assumed *k*_*p,V*_ was assigned as a global parameter (uniform throughout the domain). In a previous study, this model was selected as the model that best balances model fit and model complexity [25]. Second, we considered the case where *k*_*p,V*_ was assigned locally within the tumor. With these two additional parameterization approaches, we generated a total of 10 models to calibrate per animal. For the locally varying approach, parameter values were calibrated within the tumor at a subset of the points and then interpolated elsewhere. For example, for a given 3 × 3 voxel sub-region within the tumor ROI, the parameter values were calibrated at the corner and center positions, while the remaining four points were interpolated from the nearest calibrated values. This parameter calibration approach both significantly reduced the number of individual parameters that needed to be calibrated while also smoothing or regularizing the parameter field spatially. For models *C*_*2*_ and *C*_*4*_, which incorporate the LQ model of response to radiation therapy, *α*_*I*_ and *α*_*LT*_ are both assigned to 1 and the LQ parameter *α* is calibrated. The parameter *β* is calculated assuming an *α*/*β* ratio of 14 (assigned from literature values [42]). While the calibration approach (Fig. 1b) is briefly discussed here, the interested reader is referred to previous studies [25, 34] for a more complete description on the algorithm used. To personalize model predictions for individual animals, model parameters were calibrated on an animal specific basis using data from time points 2 to 5 via a hybrid simulated-annealing Levenberg-Marquardt algorithm [25]. Briefly, an initial guess of model parameters and the initial conditions at *t*_*1*_, ${\phi}_T\left(\overline{x},{t}_1\right)$ and ${\phi}_V\left(\overline{x},{t}_1\right)$, are used to return estimates of ${\phi}_T\left(\overline{x},{t}_2\to {t}_5\right)$ and ${\phi}_V\left(\overline{x},{t}_2\to {t}_5\right)$ via a finite difference simulation of the model system. Then, a Jacobian is numerically determined and used to determine the appropriate change in model parameters to decrease the objective function (simply, the sum of the squared errors between each of the measured and simulated ${\phi}_T\left(\overline{x},t\right)$ and ${\phi}_V\left(\overline{x},t\right)$ values). A standard Levenberg-Marquardt algorithm typically only accepts changes in model parameters that result in a decrease in the objective function. Here, we use a simulated-annealing approach to accept changes in model parameters that result in a decrease in the objective function value and occasionally accept increases in the objective function based off of the simulated annealing criterion. By incorporating a stochastic element, this hybrid algorithm allows this approach to escape potential local minimum and find the global minimum. The algorithm ceases when either the error in the objective function stagnates (less than 0.5% change in successive iterations) or when 1000 iterations are reached.

### Model selection

We utilized the Akaike Information criterion (AIC [43]) to select the model (Fig. 1c) that optimally balances model complexity and model-data agreement. The AIC is defined as:

10 $$ AIC=2k+n\ln \left(\frac{RSS}{n}\right)+2k\left(\frac{k+1}{n-k-1}\right) $$

where *k* is equal to the number of parameters calibrated for a given model, *n* is the number of data points used to calibrate the model, and *RSS* is the residual sum squares between the measured and model ${\phi}_T\left(\overline{x},t\right)$ and ${\phi}_V\left(\overline{x},t\right)$. For each animal, we calculated the AIC for each model and ranked the models from 1 to 10. We then calculated the average rank for each model and selected the model with the lowest average rank.

### Model prediction and error analysis

The model prediction step is summarized in Fig. 1d. We considered two models in the prediction stage: (1) the lowest ranked model and (2) the highest ranked model. The calibrated model parameters were used to simulate each model forward in time to predict tumor growth at the remaining time points not used in the model calibration (*t*_*6*_ and *t*_*7*_). The model predicted ${\phi}_T\left(\overline{x},t\right)$ and ${\phi}_V\left(\overline{x},t\right)$ were compared directly to the measured ${\phi}_{T, meas}\left(\overline{x},t\right)$ and ${\phi}_{V, meas}\left(\overline{x},t\right)$ at the global and local levels. At the global level, we calculated the percent error in predicted tumor volume and the Dice coefficient. The Dice coefficient describes the degree of overlap of the predicted and measured tumor volumes with a Dice value of 1 indicating perfect overlap. At the local level we calculated the concordance correlation coefficient (CCC) which describes the level of agreement between the predicted and measured values at each voxel location.

The Lilliefors test (‘*lillietest*’ function in MATLAB) was used to determine if the set of error observations from each time point, model, and error metric comes from a distribution in the normal family. No signficant *p* values (at a 5% significance level) were observed; thus we are unable to reject the null hypothesis that the observations come from a distribution in the normal family. All results are presented as the mean and 95% confidence interval when appropriate. A two-sample t-test for distrubutions with equal means and variances was used to evaluate the differences between the two separate model predictions. A *p*-value of < 0.05 was considered significant.

## Results

### Model selection

Table 3 shows the results of the model selection process. The *C*_*3*_ radiation therapy coupled (or vasculature coupled) approach with global parameters had the lowest average rank (2.62) and was tied with the *C*_*4*_ (or OER coupled) approach as the most frequently selected model (5 out of 13). Additionally, the *C*_*2*_ radiation therapy coupled (or LQ coupled) approach was selected for 3 out of 13 animals. The *C*_*1*_ radiation therapy coupled (or logistic growth coupled) approach with local parameters had the highest average rank (9.69). In the following analysis, model *C*_*3*_ with global parameters (most selected) was compared to model *C*_*1*_ with local parameters (least selected). For the following analysis, we will abbreviate these two models as *RT1* (most selected model) and *RT2* (least selected model).

**Table 3:** Model selection results

Radiation therapy coupling model	Global parameters	Local parameters
*C*_*1*_	6.85 (0)	9.69 (0)
*C*_*2*_	3.08 (3)	6.77 (0)
*C*_*3*_	^α^2.62 (5)	6.92 (0)
*C*_*4*_	2.85 (5)	7.38 (0)
*C*_*5*_	3.08 (0)	5.77 (0)

Average rank (number of animals ranked first). ^α^ indicates lowest score

### Model prediction

**Fig. 2:**
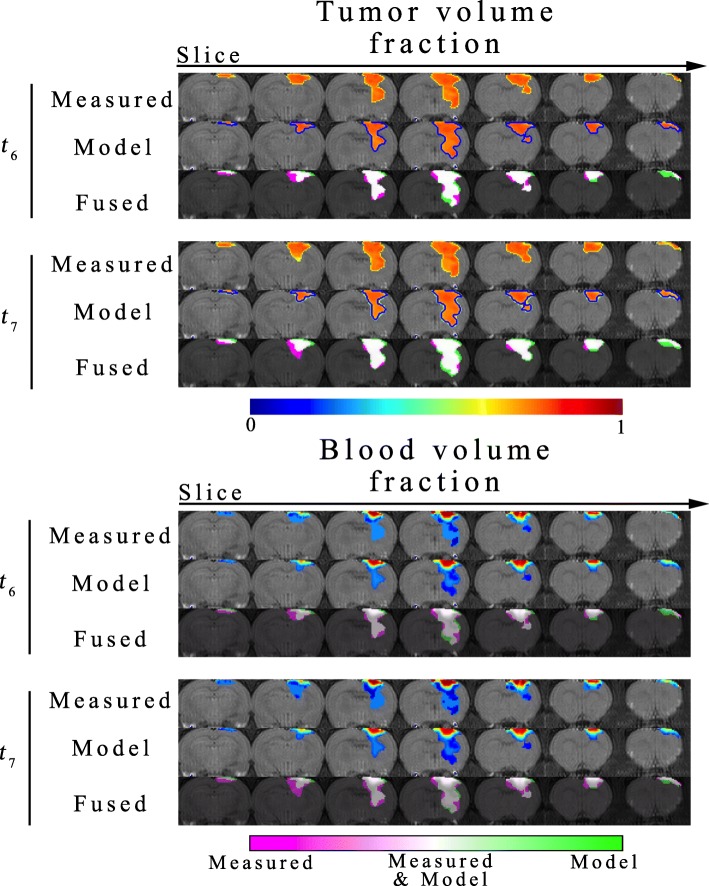
Predicted response to radiation therapy for a representative animal receiving 20 Gy. Results are shown over the entire tumor volume for a representative animal receiving a single fraction of 20 Gy. The measured and model predicted ${\phi}_T\left(\overline{x},t\right)$ and ${\phi}_V\left(\overline{x},t\right)$ are shown for the *RT1* model at *t*_*6*_ and *t*_*7*_, respectively. The fused image depicts areas where there exists perfect agreement between the model and measurement (white areas), where the model exists where the measurement does not (green areas), and where the measurement exists and the model does not (pink areas). The blue contours represent the prediction of the *RT2* model. The distributions of ${\phi}_T\left(\overline{x},t\right)$ and ${\phi}_V\left(\overline{x},t\right)$ are normalized to the maximum value of ${\phi}_T\left(\overline{x},t\right)$ and ${\phi}_V\left(\overline{x},t\right)$ observed in the measurement, and thus share a colorbar ranging from 0 to 1. This particular animal has a relatively spatially homogenous distribution of tumor cells. That is, it appears that no necrosis or low cell density regions have developed despite, poorly vascularized regions observed on the measured ${\phi}_V\left(\overline{x},t\right)$. The *RT2* model overestimates tumor growth most noticeably in slices 1, 5, 6, and 7. Visually, the *RT1* model resulted in accurate predictions of low and high ${\phi}_V\left(\overline{x},t\right)$ regions at both *t*_*6*_ and *t*_*7*_. The highest level agreement (white areas) was observed for the high ${\phi}_V\left(\overline{x},t\right)$ region near the brain-skull boundary. Less agreement regions were observed away from the brain-skull boundary

**Fig. 3:**
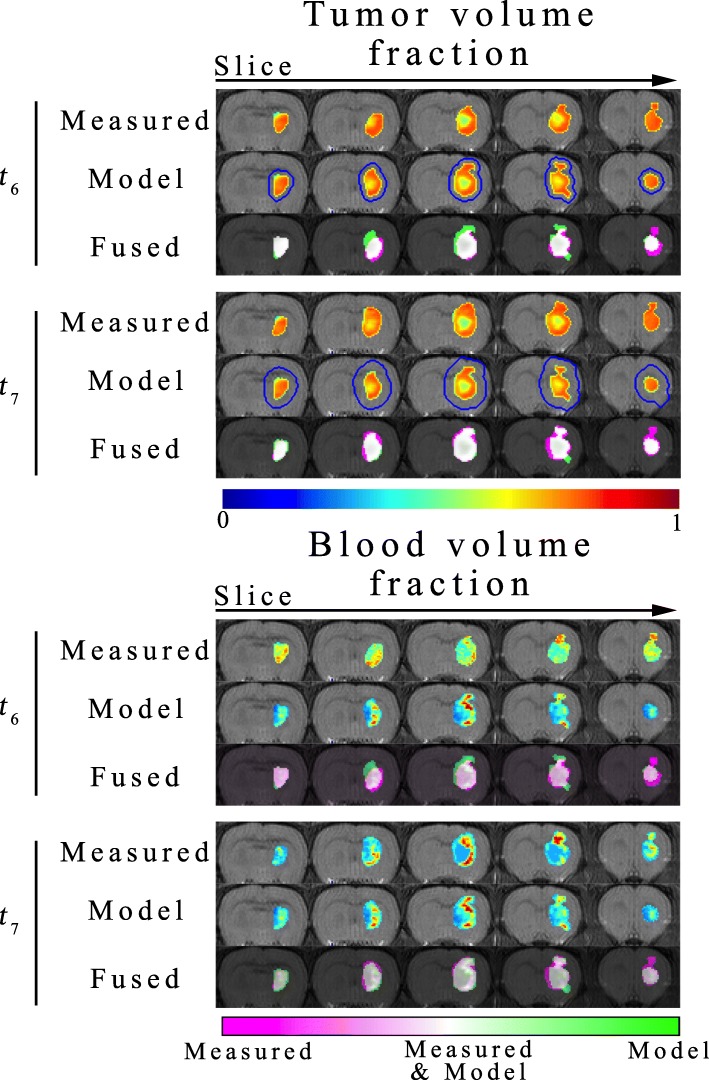
Predicted response to radiation therapy for a representative animal receiving 40 Gy. Results are shown over the entire tumor volume for a representative animal receiving a single fraction of 40 Gy. (The results are presented in an identical fashion to Fig. 2) The *RT1* model is capable of predicting the spatial heterogeneity (the development of necrosis) observed in the measured ${\phi}_T\left(\overline{x},t\right)$. The *RT2* model overestimates the tumor volume at both prediction time points. For ${\phi}_V\left(\overline{x},t\right)$, the *RT1* model was able to predict areas of low ${\phi}_V\left(\overline{x},t\right)$ in the interior and areas of high ${\phi}_V\left(\overline{x},t\right)$ at the periphery of the tumor. The model, however, predicts a lower ${\phi}_V\left(\overline{x},t\right)$ at the interior of the tumor in comparison to the measurement. The fused images demonstrate a high level of agreement in the tumor (white regions), as well as areas where the model fails to predict future tumor growth extent (pink regions)

**Fig. 4:**
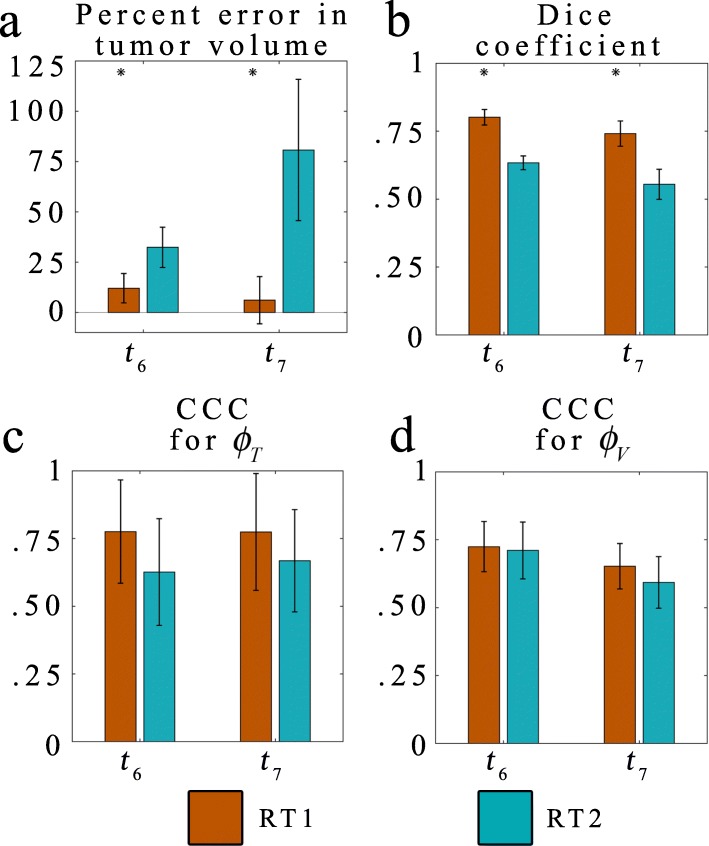
Summary statistics for the entire animal cohort. The summary statistics for the *RT1* (orange bars) and *RT2* (blue bars) are shown for both ${\phi}_T\left(\overline{x},t\right)$ and ${\phi}_V\left(\overline{x},t\right)$ predictions. Error bars represent the 95% confidence interval. Panel **a** shows the percent error in tumor volume at *t*_*6*_ and *t*_*7*_. For the *RT1* and *RT2* models error ranged from 6.11 to 80.69%. Dice coefficients (panel **b**) generally decreased overtime with values ranging from 0.55 to 0.80. At the local level, the agreement was assessed using the CCC, resulting in values ranging from 0.63 to 0.78 for ${\phi}_T\left(\overline{x},t\right)$ predictions (panel **c**), and 0.59 to 0.73 for ${\phi}_V\left(\overline{x},t\right)$ predictions (panel **d**). Statistical significance (*p* < 0.05) between models are indicated by ‘*’

Figures 2, 3 and 4 report the results of the model prediction phase. Figure 2 shows both the predicted and measured ${\phi}_T\left(\overline{x},t\right)$ and ${\phi}_V\left(\overline{x},t\right)$ over the entire tumor volume for a representative animal receiving a single fraction of 20 Gy. The distributions for ${\phi}_T\left(\overline{x},t\right)$ and ${\phi}_V\left(\overline{x},t\right)$ are also shown for *RT1*, while the white contours indicate the *RT2* prediction on the ${\phi}_T\left(\overline{x},t\right)$ images. For this particular animal, no clear necrosis (low cell density region) is observed in the post-treatment time points. High ${\phi}_V\left(\overline{x},t\right)$ is observed near the brain-skull interface, while it decreases towards the interior of the brain. Visually, there appears to be a strong agreement in the overall shape and distribution of ${\phi}_T\left(\overline{x},t\right)$ and ${\phi}_V\left(\overline{x},t\right)$ at both *t*_*6*_ and *t*_*7*_. The fused image showing the differences between the measured and predicted distributions indicate that there are some areas that the model incorrectly predicts tumor cells where there are none (bright green regions). Likewise, there are areas where the model fails to predict tumor cells where they exist in the data (bright pink regions). These visual observations are confirmed through the percent error in tumor volume, Dice coefficient, and CCC reported below. Both *RT1* and *RT2* models have less than 6% error in the predicted tumor volume at *t*_*6*_. A high degree of overlap is found between the predicted and observed tumor volume with Dice coefficients of 0.83 and 0.82 for the *RT1* and *RT2* models, respectively. However, at the voxel level, the *RT1* model has a strong level of agreement to the measured tumor cell fraction with a CCC of 0.76, while the *RT2* model has a CCC of 0.54. At *t*_*7*_, less than 20% error is observed in tumor volume predictions for the *RT1* and *RT2* models. A high degree of overlap still exists between the predicted and measured tumor volumes resulting in Dice correlation coefficients of 0.81 and 0.79 for the *RT1* and *RT2* model, respectively. At the local level, the *RT1* and *RT2* models have CCCs of 0.70 and 0.44, respectively. For ${\phi}_V\left(\overline{x},t\right)$, CCCs greater than 0.95 are observed for the *RT1* model at both *t*_*6*_ and *t*_*7*_, while CCCs less than 0.10 are observed for the *RT2* model.

Similar to Fig. 2, Fig. 3 depicts a representative animal receiving a single fraction of 40 Gy. Unlike the animal in Fig. 2, this animal appears to have developed necrosis (or low ${\phi}_T\left(\overline{x},t\right)$ regions) in the prediction time points. Generally, these low ${\phi}_T\left(\overline{x},t\right)$ correspond to low ${\phi}_V\left(\overline{x},t\right)$ regions in the right hand panels. Both the model predictions for ${\phi}_T\left(\overline{x},t\right)$ and ${\phi}_V\left(\overline{x},t\right)$ appear to recapitulate this phenonoma resulting in increased ${\phi}_T\left(\overline{x},t\right)$ and ${\phi}_V\left(\overline{x},t\right)$ at the periphery and decreased ${\phi}_T\left(\overline{x},t\right)$ and ${\phi}_V\left(\overline{x},t\right)$ towards the interior of the tumor. Additionally, compared to the 20 Gy animal, the *RT2* model seems to have increased overestimation of the future tumor growth. These qualitative observations are supported by the quantitative error assessment. For example, the *RT1* model resulted in less than 2% error in tumor volume predictions compared to the *RT2* model which has greater than 113% error in tumor volume predictions. The high degree of overlap of the predicted and observed tumor volumes for the *RT1* model is reflected in a Dice correlation coefficient of 0.85, while the *RT2* model has a value of 0.50. At the local level, *RT1* and *RT2* models have CCCs of 0.68 and 0.01, respectively. Similarly, for *t*_*7*_*,* the *RT1* model resulted in less than 12% error in tumor volume predictions compared to the *RT2* model which has greater than 193% error in predictions. A high degree of overlap between the predicted and observed tumor volume is observed resulting in Dice correlation coefficients of 0.83 and 0.52 for the *RT1* and *RT2* models, respectively. At the local level, the *RT1* model has provided accurate descriptions of the intratumoral heterogeneity resulting in CCCs of 0.70, while the *RT2* model resulted in CCCs of 0.05. For ${\phi}_V\left(\overline{x},t\right)$, CCCs greater than 0.62 are observed for the *RT1* model at both *t*_*6*_ and *t*_*7*_, while CCCs less than 0.37 are observed for the *RT2* model for the same time points.

Fig. 4 summarizes global and local error analysis for the cohort. The *RT1* model has less than 12% error in tumor volume predictions (panel a) at both *t*_*6*_ and *t*_*7*_, wheras the *RT2* model has greater than 32% error. Statistically significant (*p* < 0.05) differences are observed between the *RT1* and *RT2* model at *t*_*6*_ and *t*_*7*_. A high level of spatial agreement is observed between the predicted and observed tumor volumes for the *RT1* model resulting in Dice coefficients (panel b) greater than 0.74 at both timepoints. Lower agreement is observed for the *RT2* model, which resulted in Dice coefficients less than 0.63 at both time points. The *RT1* model resulted in statistically significant (*p* < 0.05) larger Dice coefficients compared to the *RT2* model. At the local level, the *RT1* model has a high level of agreement between the predicted and measured ${\phi}_T\left(\overline{x},t\right)$ with CCCs (panel c) greater than 0.77 for both *t*_*6*_ and *t*_*7*_. Similarily, the *RT2* has a modest level of agreemennt with CCCs greater greater than 0.63 for both *t*_*6*_ and *t*_*7*_. For the blood volume fraction predictions (panel d), a modest level of agreement is observed between the predicted and observed ${\phi}_V\left(\overline{x},t\right)$ for the *RT1* model resulting in CCCs greater than 0.65 at both time points. The *RT2* model performed nearly as well as the *RT1* model resulting in CCCs greater than 0.59.

## Discussion

Ten biologically-based models of tumor and vasculature response to radiation therapy were developed and tested for their ability to capture variations in the individual animal responses in radiation response. Each model was calibrated for individual animals using serial DW- and DCE-MRI estimates of tumor and blood volume fractions. The model that coupled radiation response to tumor vascularity using all global model parameters (*C*_*3*_) was selected as the model that optimally balanced model complexity and model fit. This selected model was then used to provide a forecast of the 3D distribution of both tumor and blood volume fractions for each animal. The selected model resulted in low global level errors (percent error in tumor volume < 12%, Dice coefficients > 0.74) and local level errors (CCCs > 0.65) for tumor and blood volume fractions predictions. This study illustrates the ability of this image-driven, subject-specific modeling framework to capture and describe tumor-specific response to whole brain radiation therapy. Notably, the two most selected models (*C*_*3*_ and *C*_*4*_) both coupled radiation therapy efficacy to vascular distribution.

The varied response of glioma (and other solid tumors) to radiation therapy is linked to the spatial and biological heterogeneity unique to each tumor. Notably in the case of brain tumors [44], heterogeneity in hypoxia status is associated with tumor progression and overall survival due to increased resistance to radiation therapy. Several emerging imaging [45, 46] methods such as ^18^F-fluoromisonidazole positron emission tomography (^18^F-MISO PET) and oxygen enhanced MRI can quantify the extent of hypoxia pre-treatment potentially providing the means to optimize therapy for individual tumors. Using ^18^F-MISO PET data, Rockne et al. demonstrated improved characterization of individual patient radiosensitivity compared to approaches that ignored the effect of hypoxia on treatment sensitivity. However, the dynamics of hypoxia (and thus radiosensitivity) is likely to vary during treatment due to the phenomenon of reoxygenation during radiation therapy [47]. A recent modeling study by Alfonso et al. [48] also demonstrated in silico the importance of characterizing radiosensitivity dynamics during the course of fractionated radiation therapy. Specifically, the initial distribution of intratumoral radiosensitivity and the fractionation scheme significantly impact the efficacy or (overall radiosensitivity) during the course of fractionated therapy. Therefore, to adapt radiation therapy to counteract the temporal and spatial variations in radiosensitivity, characterization of the upstream processes that lead to hypoxia or changes in radiosensitivity are needed. There have been several attempts at describing these dynamics at the volume-level [49, 50] and with cell scale models [51]; however, to adapt therapy to potentially address spatial variability in radiosensitivity, a spatially and temporally resolved tumor-specific mathematical description is required. In this effort, we developed and systematically characterized a spatiotemporal model of tumor and vasculature response to radiation therapy. It is the inability of the local vasculature to support local tumor cell growth that leads to spatial variations in hypoxia. To the best of our knowledge, this is the first effort to characterize the tumor and vasculature response to radiation therapy temporally and spatially using imaging driven mathematical models.

There are several areas for further development of our experimental-computational approach. First, a limitation we have previously discussed in detail elsewhere [25] is the use of DW-MRI to estimate tumor volume fraction. While the techniques has been well studied in both the pre-clinical and clinical settings and appears to be well-suited for characterizing tumor properties [10], the *ADC* does provide only a first-order approximation of true cell density. Second, we acknowledge that the doses of radiation therapy used in this study exceed what is commonly given clinically in a single instance. Nevertheless, in the pre-clinical setting, large single fraction doses are commonly used to exhibit a strong distinct response between treatment groups. Clinically, large doses of radiation are delivered typically in 2 Gy fractions to maximize the therapeutic effect and minimize damage to healthy tissue. Additionally, fractionated therapy presents the opportunity to target populations of cells that have become more radiosensitivity following reoxygenation. Furthermore, ongoing efforts are focused on applying this modeling approach to more clinically relevant fractionated therapy in a cohort of animals [52]. Third, the mechanical tissue properties are temporally invariant and literature assigned, and the linear relationship between vascularity and carrying capacity may not reflect the true in vivo or clinical scenario. Finally, we acknowledge that the current implementation uses more time points for model calibration than is commonly acquired in the clinical setting. However, MRI-guided external beam devices could foreseeably acquire the necessitated quantitative and anatomical image images before and during treatment for model calibration [53, 54].

There are several points of consideration for translating these efforts to the clinical setting. One important difference in tumor biology between the pre-clinical C6 model and high grade gliomas is the presence of both enhancing (clinical disease) and non-enhancing (sub-clinical or invasive disease) tumor regions [55]. Our current approach could be applied to the enhancing tumor region as it is most similar to what is observed in the C6 murine model of glioma. However, it is less clear how cell density varies throughout the non-enhancing region, and thus assigning cell density is difficult. One solution is to apply fixed cell density values within this region as is done in [56]. Second, high grade glioma patients typically receive surgery, followed by fractionated radiation therapy and temozolomide [57], as opposed to the single fraction radiotherapy used in this study. As currently formulated, the model does not consider these elements; however, it could be easily incorporated by applying Eq. (6) every time radiation therapy is delivered or by incorporating the cytotoxic effects of the systemic therapy into Eq. (7), as we have done clinical breast cancer setting [39]. Third, there is the computational intensity of this approach. For a single subject, calibration of the most-complex model is feasible on a personal computer. However, for multiple model calibrations and multiple subjects (in this paper we had 130 individual calibrations) it is more efficient to utilize multiple compute nodes on a high-performance computing system. For reference, a single calibration took less than 5 h on a personal computer (4 GHz Intel Core i7-6700K with 4 cores), or less than 3 on a compute node (2.6 GHz Intel Xeon E5–2690 v3 with 12 cores). A single simulation time step takes ~ 0.3 s. In the clinical setting, medical images are typically collected with a larger sampling matrix (512^2^ or 256^2^ vs 128^2^), thus leading to a larger simulation domain (and therefore greater computational intensity). In preliminary clinical efforts, a single simulation time step on the larger simulation domain now takes ~ 0.8 s. Finally, at the clinical level there is the additional opportunity to incorporate additional biological information (e.g., immunohistochemistry analysis, vascular or cellular density) into the model.

## Conclusions

We have developed and applied a biologically-based, mathematical model of tumor and vasculature response to radiation therapy that can be parameterized for individual tumors using non-invasive quantitative imaging measures. Importantly, we have demonstrated an experimental-computational framework to non-invasively and spatially resolve response dynamics for individual animals that can be used to forecast future response. Notably, the most selected model described the 3D dynamics of tumor and blood volume fraction and linked tumor vasculature to spatial radiation response. Future efforts should include expanding this modeling framework to the setting of fractionated pre-clinical and clinical data.

## Data Availability

The datasets generated and/or analyzed during the current study are available from the corresponding author on reasonable request.

## Abbreviations

ADC: Apparent diffusion coefficient
AIC: Akaike information criterion
CCC: Concordance correlation coefficient
DCE-MRI: Dynamic contrast-enhanced
DW-MRI: Diffusion-weighted
LQ: Linear quadratic
MRI: Magnetic resonance imaging
OER: Oxygen enhancement ratio
ROI: Regions of interest
RSS: Residual sum squared error
RT1: Most selected radiation therapy model
RT2: Least selected radiation therapy model

## References

[1] Connell PP, Hellman S (2009). Advances in radiotherapy and implications for the next century: a historical perspective. Cancer Res.

[2] Omuro A, DeAngelis LM (2013). Glioblastoma and other malignant gliomas: a clinical review. JAMA.

[3] Giese A, Bjerkvig R, Berens ME, Westphal M (2003). Cost of migration: invasion of malignant Gliomas and implications for treatment. J Clin Oncol.

[4] Jones KM, Michel KA, Bankson JA, Fuller CD, Klopp AH, Venkatesan AM (2018). Emerging magnetic resonance imaging Technologies for Radiation Therapy Planning and Response Assessment. Int J Radiat Oncol Biol Phys.

[5] Hormuth DA, Weis JA, Barnes S, Miga MI, Quaranta V, Yankeelov TE (2018). Biophysical Modeling of In Vivo Glioma Response After Whole-Brain Radiation Therapy in a Murine Model of Brain Cancer. Int J Radiat Oncol.

[6] Rockne RC, Trister AD, Jacobs J, Hawkins-Daarud AJ, Neal ML, Hendrickson K, Mrugala MM, Rockhill JK, Kinahan P, Krohn KA (2015). A patient-specific computational model of hypoxia-modulated radiation resistance in glioblastoma using (18)F-FMISO-PET. J R Soc Interface.

[7] Sunassee ED, Tan D, Ji N, Brady R, Moros EG, Caudell JJ, Yartsev S, Enderling H (2019). Proliferation saturation index in an adaptive Bayesian approach to predict patient-specific radiotherapy responses. Int J Radiat Biol.

[8] Prokopiou S, Moros EG, Poleszczuk J, Caudell J, Torres-Roca JF, Latifi K, Lee JK, Myerson R, Harrison LB, Enderling H (2015). A proliferation saturation index to predict radiation response and personalize radiotherapy fractionation. Radiat Oncol.

[9] Wen PY, Macdonald DR, Reardon DA, Cloughesy TF, Sorensen AG, Galanis E, DeGroot J, Wick W, Gilbert MR, Lassman AB (2010). Updated response assessment criteria for high-grade Gliomas: response assessment in Neuro-oncology working group. J Clin Oncol.

[10] Hormuth DA, Sorace AG, Virostko J, Abramson RG, Bhujwalla ZM, Enriquez-Navas P, Gillies R, Hazle JD, Mason RP, Quarles CC (2019). Translating preclinical MRI methods to clinical oncology. J Magn Reson Imaging.

[11] Hamstra DA, Galban CJ, Meyer CR, Johnson TD, Sundgren PC, Tsien C, Lawrence TS, Junck L, Ross DJ, Rehemtulla A (2008). Functional diffusion map as an early imaging biomarker for high-grade glioma: correlation with conventional radiologic response and overall survival. J Clin Oncol.

[12] Zhang J, Liu H, Tong H, Wang S, Yang Y, Liu G, Zhang W (2017). Clinical applications of contrast-enhanced perfusion MRI techniques in Gliomas: recent advances and current challenges. Contrast Media Mol Imaging.

[13] Tsien C, Cao Y, Chenevert T (2014). Clinical applications for diffusion magnetic resonance imaging in radiotherapy. Semin Radiat Oncol.

[14] Cao Y (2011). The promise of dynamic contrast-enhanced imaging in radiation therapy. Semin Radiat Oncol.

[15] Li X, Abramson RG, Arlinghaus LR, Kang H, Chakravarthy AB, Abramson VG, Farley J, Mayer IA, Kelley MC, Meszoely IM (2015). Multiparametric magnetic resonance imaging for predicting pathological response after the first cycle of Neoadjuvant chemotherapy in breast Cancer. Invest Radiol.

[16] Roque T, Risser L, Kersemans V, Smart S, Allen D, Kinchesh P, Gilchrist S, Gomes AL, Schnabel JA, Chappell MA (2017). A DCE-MRI driven 3-D reaction-diffusion model of solid tumour growth. IEEE Trans Med Imaging.

[17] Hawkins-Daarud A, Rockne RC, Anderson ARA, Swanson KR (2013). Modeling tumor-associated edema in gliomas during anti-angiogenic therapy and its impact on imageable tumor. Front Oncol.

[18] Gaw N, Hawkins-Daarud A, Hu LS, Yoon H, Wang L, Xu Y, Jackson PR, Singleton KW, Baxter LC, Eschbacher J (2019). Integration of machine learning and mechanistic models accurately predicts variation in cell density of glioblastoma using multiparametric MRI. Sci Rep.

[19] Swan A, Hillen T, Bowman JC, Murtha AD (2018). A patient-specific anisotropic diffusion model for brain tumour spread. Bull Math Biol.

[20] Lipková J, Angelikopoulos P, Wu S, Alberts E, Wiestler B, Diehl C, Preibisch C, Pyka T, Combs SE, Hadjidoukas P (2019). Personalized radiotherapy Design for Glioblastoma: integrating mathematical tumor models, multimodal scans, and Bayesian inference. IEEE Trans Med Imaging.

[21] Unkelbach J, Menze B, Konukoglu E, Dittmann F, Ayache N, Shih HA (2014). Radiotherapy planning for glioblastoma based on a tumor growth model: implications for spatial dose redistribution. Phys Med Biol.

[22] Rockne RC, Hawkins-Daarud A, Swanson KR, Sluka JP, Glazier JA, Macklin P, Hormuth DA, Jarrett AM, Lima EABF, Tinsley Oden J (2019). The 2019 mathematical oncology roadmap. Phys Biol.

[23] Hormuth DA, Weis JA, Barnes SL, Miga MI, Rericha EC, Quaranta V, Yankeelov TE (2015). Predicting in vivo glioma growth with the reaction diffusion equation constrained by quantitative magnetic resonance imaging data. Phys Biol.

[24] Hormuth DA, Weis JA, Barnes SL, Miga MI, Rericha EC, Quaranta V, Yankeelov TE (2017). A mechanically coupled reaction–diffusion model that incorporates intra-tumoural heterogeneity to predict in vivo glioma growth. J R Soc Interface.

[25] Hormuth DA, Jarrett AM, Feng X, Yankeelov TE (2019). Calibrating a predictive model of tumor growth and angiogenesis with quantitative MRI. Ann Biomed Eng.

[26] Yankeelov TE, Atuegwu N, Hormuth DA, Weis JA, Barnes SL, Miga MI, Rericha EC, Quaranta V (2013). Clinically relevant modeling of tumor growth and treatment response. Sci Transl Med.

[27] Douglas BG, Fowler JF (1976). The effect of multiple small doses of x rays on skin reactions in the mouse and a basic interpretation. Radiat Res.

[28] Grassberger C, Paganetti H (2016). Methodologies in the modeling of combined chemo-radiation treatments. Phys Med Biol.

[29] Hormuth D, Jarrett A, Lima E, McKenna M, Fuentes D, Yankeelov T (2019). Mechanism-based modeling of tumor growth and treatment response constrained by multiparametric imaging data. J Clin Oncol Clin Cancer Inform.

[30] Jarrett AM, Lima EABF, Hormuth DA, McKenna MT, Feng X, Ekrut DA, Resende ACM, Brock A, Yankeelov TE (2018). Mathematical models of tumor cell proliferation: a review of the literature. Expert Rev Anticancer Ther.

[31] Brüningk S, Powathil G, Ziegenhein P, Ijaz J, Rivens I, Nill S, Chaplain M, Oelfke U, ter Haar G (2018). Combining radiation with hyperthermia: a multiscale model informed by in vitro experiments. J R Soc Interface.

[32] Rutter EM, Stepien TL, Anderies BJ, Plasencia JD, Woolf EC, Scheck AC, Turner GH, Liu Q, Frakes D, Kodibagkar V (2017). Mathematical analysis of Glioma growth in a murine model. Sci Rep.

[33] Massey SC, Rockne RC, Hawkins-Daarud A, Gallaher J, Anderson ARA, Canoll P, Swanson KR (2018). Simulating PDGF-driven Glioma growth and invasion in an anatomically accurate brain domain. Bull Math Biol.

[34] Hormuth D, Eldridge SB, Weis J, Miga MI, Yankeelov TE, von Stechow L (2018). Mechanically coupled reaction-diffusion model to predict Glioma growth: methodological details. Springer methods and protocols: Cancer systems biology.

[35] Eriksson D, Stigbrand T (2010). Radiation-induced cell death mechanisms. Tumor Biol.

[36] Baskar R, Dai J, Wenlong N, Yeo R, Yeoh K-W (2014). Biological response of cancer cells to radiation treatment. Front Mol Biosci.

[37] Whisenant JG, Ayers GD, Loveless ME, Barnes SL, Colvin DC, Yankeelov TE (2014). Assessing reproducibility of diffusion-weighted magnetic resonance imaging studies in a murine model of HER2+ breast cancer. Magn Reson Imaging.

[38] Atuegwu NC, Colvin DC, Loveless ME, Xu L, Gore JC, Yankeelov TE (2012). Incorporation of diffusion-weighted magnetic resonance imaging data into a simple mathematical model of tumor growth. Phys Med Biol.

[39] Jarrett A, Hormuth D, Barnes S, Feng X, Huang W, Yankeelov T (2018). Incorporating drug delivery into an imaging-driven, mechanics-coupled reaction diffusion model for predicting the response of breast cancer to neoadjuvant chemotherapy: theory and preliminary clinical results. Phys Med Biol.

[40] Weis JA, Miga MI, Arlinghaus LR, Li X, Abramson V, Chakravarthy AB, Pendyala P, Yankeelov TE (2015). Predicting the response of breast Cancer to Neoadjuvant therapy using a mechanically coupled reaction-diffusion model. Cancer Res.

[41] Hormuth DA, Skinner JT, Does MD, Yankeelov TE (2014). A comparison of individual and population-derived vascular input functions for quantitative DCE-MRI in rats. Magn Reson Imaging.

[42] Stuschke M, Budach V, Budach W, Feldmann HJ, Sack H (1992). Radioresponsiveness, sublethal damage repair and stem cell rate in spheroids from three human tumor lines: comparison with xenograft data. Int J Radiat Oncol Biol Phys.

[43] Akaike H (1974). A new look at the statistical model identification. Automatic Control IEEE Transact.

[44] Jensen RL (2009). Brain tumor hypoxia: tumorigenesis, angiogenesis, imaging, pseudoprogression, and as a therapeutic target. J Neurooncol.

[45] Padhani AR, Krohn KA, Lewis JS, Alber M (2007). Imaging oxygenation of human tumours. Eur Radiol.

[46] Salem Ahmed, Little Ross A., Latif Ayşe, Featherstone Adam K., Babur Muhammad, Peset Isabel, Cheung Susan, Watson Yvonne, Tessyman Victoria, Mistry Hitesh, Ashton Garry, Behan Caron, Matthews Julian C., Asselin Marie-Claude, Bristow Robert G., Jackson Alan, Parker Geoff J.M., Faivre-Finn Corinne, Williams Kaye J., O'Connor James P.B. (2019). Oxygen-enhanced MRI Is Feasible, Repeatable, and Detects Radiotherapy-induced Change in Hypoxia in Xenograft Models and in Patients with Non–small Cell Lung Cancer. Clinical Cancer Research.

[47] Kallman RF, Dorie MJ (1986). Tumor oxygenation and reoxygenation during radiation theraphy: Their importance in predicting tumor response. Int J Radiat Oncol.

[48] Alfonso JCL, Berk L (2019). Modeling the effect of intratumoral heterogeneity of radiosensitivity on tumor response over the course of fractionated radiation therapy. Radiat Oncol.

[49] Sachs RK, Hlatky LR, Hahnfeldt P (2001). Simple ODE models of tumor growth and anti-angiogenic or radiation treatment. Math Comput Model.

[50] Belfatto A, Riboldi M, Ciardo D, Cattani F, Cecconi A, Lazzari R, Jereczek-Fossa BA, Orecchia R, Baroni G, Cerveri P (2016). Modeling the interplay between tumor volume regression and oxygenation in uterine cervical Cancer during radiotherapy treatment. IEEE J Biomed Heal Informatics.

[51] Powathil GG, Adamson DJA, Chaplain MAJ (2013). Towards predicting the response of a solid tumour to chemotherapy and radiotherapy treatments: clinical insights from a computational model. PLOS Comput Biol.

[52] Hormuth D, Jarret A, Wu C, Yankeelov T. Employing quantitative imaging data to personalize mathematical models of the tumor microenvironment and response to therapies. CRUK-AACR Joint Conf Eng Phys Sci Oncol. 2019. Abstract Nr. 44.

[53] Yang Y, Cao M, Sheng K, Gao Y, Chen A, Kamrava M, Lee P, Agazaryan N, Lamb J, Thomas D (2016). Longitudinal diffusion MRI for treatment response assessment: preliminary experience using an MRI-guided tri-cobalt 60 radiotherapy system. Med Phys.

[54] Corradini S, Alongi F, Andratschke N, Belka C, Boldrini L, Cellini F, Debus J, Guckenberger M, Hörner-Rieber J, Lagerwaard FJ (2019). MR-guidance in clinical reality: current treatment challenges and future perspectives. Radiat Oncol.

[55] Mabray MC, Barajas RF, Cha S (2015). Modern brain tumor imaging. The Korean brain tumor society; the Korean Society for Neuro-Oncology; The Korean Society for Pediatric Neuro-Oncology. Brain tumor Res Treat.

[56] Swanson KR, Rostomily RC, Alvord EC (2008). A mathematical modelling tool for predicting survival of individual patients following resection of glioblastoma: a proof of principle. Br J Cancer.

[57] Stupp R, Masonvan den Bent MJ, Weller M, Fisher B, Taphoorn MJB, Belanger K, Brandes AA, Marosi C, Bogdahn U, Curschmann J (2005). Radiotherapy plus Concomitant and Adjuvant Temozolomide for Glioblastoma. N Engl J Med.

